# Effects of Nutrient Fertility on Growth and Alkaloidal Content in *Mitragyna speciosa* (Kratom)

**DOI:** 10.3389/fpls.2020.597696

**Published:** 2020-12-21

**Authors:** Mengzi Zhang, Abhisheak Sharma, Francisco León, Bonnie Avery, Roger Kjelgren, Christopher R. McCurdy, Brian J. Pearson

**Affiliations:** ^1^Mid-Florida Research and Education Center, Department of Environmental Horticulture, Institute of Food and Agricultural Sciences, University of Florida, Apopka, FL, United States; ^2^Department of Pharmaceutics, College of Pharmacy, University of Florida, Gainesville, FL, United States; ^3^Department of Medicinal Chemistry, College of Pharmacy, University of Florida, Gainesville, FL, United States; ^4^Translational Drug Development Core, Clinical and Translational Science Institute, University of Florida, Gainesville, FL, United States

**Keywords:** growth, physiological response, pharmacology, medicinal plant, ketum, kakum, biak-biak

## Abstract

Leaves harvested from the Southeast Asian tree *Mitragyna speciosa* (kratom) have a history of use as a traditional ethnobotanical source of medicine to combat fatigue, improve work productivity, and to reduce opioid-related withdrawal symptoms. Kratom leaves contain an array of alkaloids thought to be responsible for the bioactivity reported by users. Interest in the consumptive effects of kratom has led to its recent popularity and use in North America, Western Europe, and Australia. Although the chemistry and pharmacology of select kratom alkaloids are understood, studies have not examined the influence of production environment on growth and alkaloidal content. To directly address this need, 68 kratom trees were vegetatively propagated from a single mother stock to reduce genetic variability and subjected to four varying fertilizer application rates. Leaves were analyzed for chlorophyll concentration, biomass, and alkaloidal content to understand the physiological response of the plant. While increasing rates of fertilizer promoted greater plant growth, relationships with alkaloidal content within leaves were highly variable. Fertility rate had little influence on the concentration of mitragynine, paynantheine, speciociliatine, mitraphylline, and corynoxine per leaf dry mass. 7-Hydroxymitragynine was below the lower limit of quantification in all the analyzed leaf samples. Low to medium rates of fertilizer, however, maximized concentrations of speciogynine, corynantheidine, and isocorynantheidine per leaf dry mass, suggesting a promotion of nitrogen allocation for secondary metabolism occurred for these select alkaloids. Strong correlations (*r*^2^ = 0.86) between extracted leaf chlorophyll and rapid, non-destructive chlorophyll evaluation (SPAD) response allowed for development of a reliable linear model that can be used to diagnose nutrient deficiencies and allow for timely adjustment of fertilization programs to more accurately manage kratom cultivation efforts. Results from this study provide a greater understanding of the concentration and synthesis of nine bioactive alkaloids in fresh kratom leaves and provide foundational information for kratom cultivation and production.

## Introduction

*Mitragyna speciosa*, also known as kratom in Thailand or ketum in Malaysia, is a facultatively deciduous small to medium size (4–16 m) tropical tree native to southern Thailand-north peninsular Malaysia, Sumatra, Borneo, Philippines, and New Guinea, but is also reported in Vietnam and Myanmar (Suwanlert, 1975; Puff, 2007). Kratom trees are found in freshwater swamp forest areas or near river systems where soil is saturated by groundwater for a duration of 8–10 months (Nilus et al., 2011). Leaves of kratom are used as a traditional, ethnobotanical medicine to provide relief of pain, fatigue, treat diarrhea, enhance work productivity, and to reduce opioid-related withdrawal symptoms (Suwanlert, 1975; Cinosi et al., 2015; Grundmann, 2017). Kratom is most often consumed by being chewed directly or steeped as a tea (Suwanlert, 1975). Interest in the application and use of kratom emerged within North America, Western Europe, and Australia in the late 20th century (NIDA, 2019). Within these newly emerged markets, kratom is sold as a concentrated liquid extract or as dried, ground leaf powder that is oftentimes encapsulated for consumptive ease. Reports of mild euphoria and increased energy resulting from consumption of kratom have led to its reported use as a recreational drug. Differences in preparation and use have likely contributed to reports of toxicity and mortality among new users in the United States and Europe. Deaths attributed to kratom use, however, are often associated with product adulteration or consumption with other, often toxic, compounds (McWhirter and Morris, 2010; Nelsen et al., 2010; Lydecker et al., 2016; Swogger and Walsh, 2018). Interestingly, no reports of mortality among kratom users have occurred in Southeast Asia where it has a long history of use. Despite growing health concerns surrounding recreational consumption of kratom, research examining medical application of kratom as a potential pharmaceutical alternative to opioid-based medication is ongoing and supported given its reported traditional use to combat opiate withdrawal (Boyer et al., 2008).

Kratom’s consumptive effects are reported to be dose-dependent, functioning primarily as either a sedative or stimulant (Suwanlert, 1975; Jansen and Prast, 1989; Cinosi et al., 2015). Low to moderate doses are reported to produce stimulant-like effects and enhance work productivity through relief of fatigue (Suwanlert, 1975; Jansen and Prast, 1989; Assanangkornchai et al., 2007; Jivan and Andurkar, 2012). Moderate to high doses are reported to produce sedative or opioid-like effects and therefore useful as a treatment for pain or diarrhea (Suwanlert, 1975; Jansen and Prast, 1989; Jivan and Andurkar, 2012). Kratom has also been reported to be used as an opium substitute and a suppressor of opioid-related withdrawal symptoms (Burkill and Haniff, 1930; Burkill, 1966; Suwanlert, 1975; Vicknasingam et al., 2010; Grundmann, 2017). However, regular kratom use was reported to be associated with drug dependency and development of withdrawal symptoms by increasing dosage and frequency of use (Suwanlert, 1975). Singh et al. (2014) found more than half of regular kratom users developed severe dependence while 45% developed moderate dependence. Withdrawal symptoms included but were not limited to skin darkness, hostility, aggression, muscle pain, difficulty sleeping, watery eyes/nose, and fever (Suwanlert, 1975; Singh et al., 2014).

Kratom contains a wide array of structurally related alkaloids and several flavonoids, terpenoid saponins, glycosides, and polyphenols (Cinosi et al., 2015), among which mitragynine and 7-hydroxymitragynine are believed to be the bioactive constituents. Mitragynine is the predominant alkaloid in kratom leaves, accounting for approximately 12 to 66% of the total alkaloid content (Ponglux et al., 1994; Takayama et al., 1998). Mitragynine produces an opioid-like antinociception effect due to the agonistic activities of the μ-, but not δ- opioid receptors by latest studies (Thongpradichote et al., 1998; Horie et al., 2005; Hassan et al., 2013; Obeng et al., 2020). This indole alkaloid also binds to α-adrenergic receptors and therefore acts as a stimulant (Maurer, 2010; McIntyre et al., 2015; Obeng et al., 2020). Sabetghadam et al. (2013) administered mitragynine at varying concentrations in rats and reported that mitragynine was relatively safe at lower sub-chronic doses (1–10 mg⋅kg^–1^) but toxic at higher doses (100 mg⋅kg^–1^). 7-Hydroxymitragynine, a minor constituent present at concentrations of up to 2% in leaves, is believed to be the major contributor to the addictive potential of kratom given its activity as a potent μ-opioid receptor agonist (Ponglux et al., 1994; Kruegel et al., 2016; Lydecker et al., 2016; Yue et al., 2018; Hemby et al., 2019). Although structurally different from morphine and produced by hydroxylation of mitragynine, 7-hydroxymitragynine resulted in tolerance of the antinociceptive effect and produced withdrawal symptoms similar to morphine when administered chronically to mice (Matsumoto et al., 2005). In a study examining electronically stimulated contraction of Dunkin-Hartley guinea pig ileum, Horie et al. (2005) found 7-hydroxymitragynine to be a 30- and 17-fold more potent agonist (pD_2_) when compared to that of mitragynine and morphine, respectively. 7-Hydroxymitragynine can cause drug-drug interactions when taken with other substances (Haron and Ismail, 2015) and therefore may be presumably responsible for reported deaths of kratom consumption, especially where drug adulteration occurred. In addition, other minor alkaloids found within leaves of kratom include paynantheine (constitute 8.9%), speciogynine (6.6%), and mitraphylline, which act as a competitive antagonist of μ-opioid receptors and function as muscle relaxants, and speciociliatine (0.8%) and corynantheidine, which act as opioid agonists (Ponglux et al., 1994; Horie et al., 2005; Assanangkornchai et al., 2007; Hassan et al., 2013; Cinosi et al., 2015; Kruegel et al., 2016). The overall effect of kratom leaves or products on human μ-opioid receptors is complex due to the interplay of bioactive alkaloids present (Kruegel and Grundmann, 2018).

A recent study examining the alkaloidal content of 6 commercially available dried and powdered, non-concentrated kratom products found mitragynine and 7-hydroxymitragynine concentrations in the range of 4.71 to 8.72% and 0.01 to 0.04%, respectively (Sharma et al., 2019). In addition, mitragynine concentrations in bulk leaf samples have been reported in previous studies, varying from 0.80 to 2.38% (Kikura-Hanajiri et al., 2009; Mudge and Brown, 2017). Sharma et al. (2019) reported corynantheidine, corynoxine, and isocorynantheidine concentrations in commercial kratom products ranged from 0.08 to 0.12%, 0.03 to 0.43%, and 0.05 to 0.13%, respectively. Ranges of paynantheine, speciociliatine, and speciogynine were relatively high with concentrations ranging from 1.81 to 3.43%, 1.36 to 4.10%, and 0.87 to 1.42%, respectively. The range of alkaloidal content found in freshly harvested, dried and powdered kratom leaves, however, is largely undocumented in literature.

**FIGURE 1:**
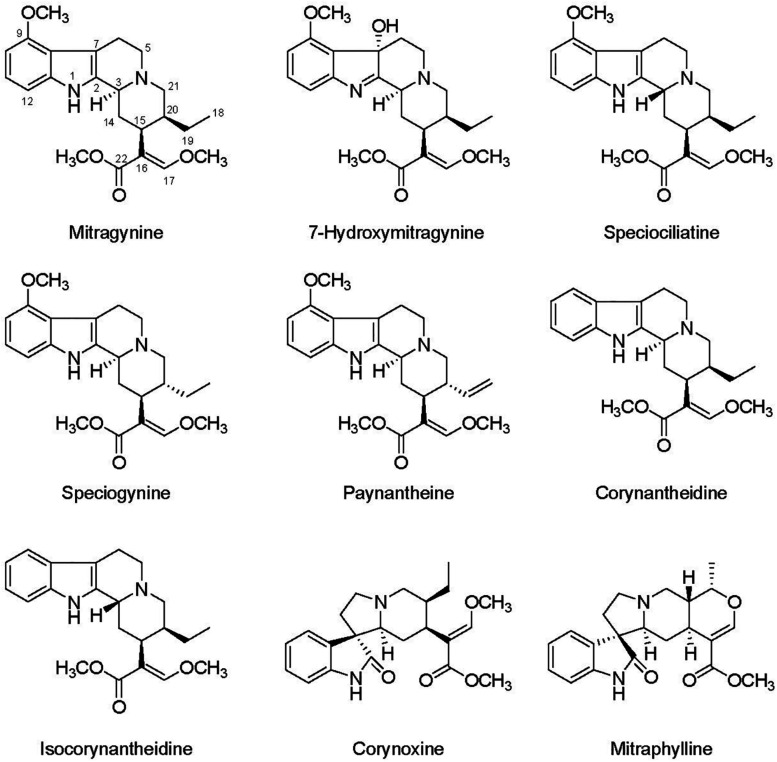
Chemical structure of selected kratom’s alkaloids.

Although alkaloids can serve to benefit human health, their primary purpose for being produced by plants is to support important ecological functions, such as in defense of pests and disease (Nchu et al., 2018). Alkaloids may be considered N-storage products accumulated under adverse environmental conditions, such as salinity and water stress, and have specific ecological functions to increase plant fitness (Höft et al., 1996; Mishra et al., 2019). The production of plant secondary metabolites depend on both endogenous and exogenous factors and nutrient fertilization is one of the most important factors to influence plant growth and production of alkaloids with high quality and quantity (Vu et al., 2006; Nchu et al., 2018). Several alkaloid-producing plants, including datura (*Datura innoxia*), tall fescue (*Festuca arundinacea*), and periwinkle (*Catharanthus roseus*), have been reported to increase alkaloid production in response to moderate or high nitrogen rates (Arechavaleta et al., 1992; Al-Humaid, 2005; Abdolzadeh et al., 2006). Despite being a high valuable medicinal plant, investigations examining the influence of production conditions on plant growth and alkaloid concentrations within kratom leaves are unavailable. This information is critical to cultivation of kratom and to better understand how production environment influences distributions of alkaloids within its leaves. To directly address this need, the specific objectives of this study were to examine the influence of nutrient application on (1) growth, yield, and chlorophyll concentrations in leaves of kratom, and (2) concentration and distribution of selected alkaloids (Figure 1) within kratom leaves. Results from this work will provide critical plant production and leaf alkaloidal content information needed by both scientists and cultivators of kratom.

## Materials and Methods

### Plant Materials

Kratom cuttings were taken from a single mother stock plant obtained from a private plant producer to reduce potential for plant genetic diversity among replicates. Stems of plant propagules were dipped into 1000 mg L^–1^ indole-3-butyric acid rooting hormone (Hormodin 1, OHP Inc., Mainland, PA, United States) and then inserted into 3.8 cm rockwool cubes (Grodan, ROXUL Inc., Milton, ON, Canada). They were placed within a laboratory at 25°C, exposed to fluorescent lighting (T5; Sunblaster, Langley, BC, Canada) for 24 h and relative humidity maintained at >50% for a duration of 6 weeks until roots were well established. Rooted propagules were transplanted into 0.7 L nursery containers filled with Fafard 4P potting media (Sun Gro Horticulture Canada Ltd., Agawam, MA, United States) containing 48% peat, 30% pine bark, 10% perlite, and 12% vermiculite. Plants were cultivated in a gutter-connected greenhouse with 30% light reducing polycarbonate paneling located in Apopka, Florida, United States (28.64°N, 81.55°W) under natural day length. Approximately 30 d after transplanting, 68 containers of the most uniform kratom trees were transplanted into larger, 11.4 L containers with soilless substrate (Pro-Line C/B Growing Mix, Jolly Gardener, Oldcastle Lawn & Garden, Inc., Atlanta, GA, United States) containing 45% Canadian sphagnum peat moss, 35% pine bark, 10% perlite, and 10% vermiculite.

### Fertilization Treatments and Environmental Conditions

Osmocote Plus 15-9-12 slow release fertilizer (Scotts, Marysville, OH, United States) containing 7% ammoniacal and 8% nitrate nitrogen, 9% phosphate and 12% soluble potash was applied to kratom replicates (*n* = 17) at either 0 (none), 46 (low), 74 (medium), or 96 g (high) per container rates. Fertilizer application rates were selected based on manufacturer’s recommendations and treatments were randomly assigned to plants. All plants were grown under natural daylight and irrigated twice a day (0600 and 1800 h) with approximately 2 L of water daily utilizing a programmable irrigation controller (Sterling 12, Superior Controls Co., Inc., Valencia, CA, United States). Greenhouse heaters and fans were controlled by an environmental control system (Wadsworth Control System, Arvada, CO, United States) and set to operate when greenhouse temperature was ≤16 or ≥23°C, respectively. Greenhouse environmental conditions were recorded every 15 min by a datalogger (WatchDog 2475; Spectrum Technologies, Inc., Aurora, IL, United States).

### Plant Measurements and Data Collection

Plant initial height (cm, measured from the substrate surface to primary meristem), width (cm), and trunk diameter (mm, measured directly above the substrate surface) were recorded approximately 1 week after plants were transplanted into 11.4 L containers (September 2018) and recorded every 4 weeks thereafter until termination of the experiment in December 2018. An index of relative chlorophyll concentration (SPAD value) was measured on leaves from the 2nd, 3rd, and 4th node on the primary stem of four randomly selected plants for each treatment using a chlorophyll meter (SPAD-502 Plus, Konica Minolta, Inc., Osaka, Japan). Three readings per leaf were measured and recorded. Total leaf number (leaf length ≥2 cm) was recorded on four randomly selected plants from each treatment. Total leaf area (cm^2^) was measured using a leaf area meter (LI-3000; LI-COR, Inc., Lincoln, NE, United States). Average leaf area (cm^2^) was calculated by dividing total leaf area by leaf number. Dry leaf mass (g) was recorded from four randomly selected plants by removing leaves, drying them in an oven at 55°C for ≥3 day, and measuring leaf mass using a laboratory balance (PL601-S, Mettler Toledo, Switzerland).

### Chlorophyll Extraction and Measurement

Three mature leaf samples were collected on the 2nd node of the primary stem of three randomly selected plants from each treatment for chlorophyll extraction once every 4 weeks from September to December 2018. The extraction procedure was performed in accordance with the modified method of Arnon et al. (1974). A 0.3 g leaf sample was homogenized (Homogenizer 850, Thermo Fisher Scientific, Inc., Waltham, MA, United States) in a test tube (13 × 100 mm Beaded Rim, Pyrex, Corning, NY, United States) with 1.5 mL of 4°C chilled 80% acetone at 15,000 rpm for 1 min. Homogenate was placed into a 1.5 mL Eppendorf tube (MCT Graduated Natural, Thermo Fisher Scientific, Inc., Waltham, MA, United States) and centrifuged (Eppendorf Centrifuge 5418, Hauppauge, NY, United States) at 10,000 rpm with a maximum g- force of 8609 × *g* for 10 min. The supernatant was transferred into a 1.5 mL plastic cuvette (Thermo Fisher Scientific, Inc., Waltham, MA, United States) and light absorbance was measured at 646, 663, and 750 nm with a UV-Visible Spectrophotometer (Evolution 201, Thermo Fisher Scientific, Inc., Waltham, MA, United States). Chl (*a* + *b*) content was calculated as:

$$\mathrm{Chl}\,\left(a+b\right)\,\left(\mu \,g/g\,\mathrm{tissue}\right)=\frac{\left(20.12*\left({\text{A}}_{646}-{\text{A}}_{750}\right)+8.02*\left({\text{A}}_{663}-{\text{A}}_{750}\right)\right)}{W}*V$$

where *V* is the volume of the acetone used for homogenization and *W* is the weight of the plant tissue used.

### Alkaloid Extraction and Measurement

Two leaves, from the 2nd and 3rd node on the primary stem of kratom, respectively, were collected on four random plants selected from each fertilizer treatment bi-weekly. Leaf samples were lyophilized and then ground into powder using a commercial grinder (KitchenAid, St. Joseph, MI, United States). Leaf powder samples (2–5 g) were extracted until exhaustion with ethanol 190 proof utilizing a slight modification of the method described by Avery et al. (2019) where the ground leaves were placed in a conical flask (20 ml) with 10 mL of ethanol, and sonicated (30°C) for 90 min, then filtered and dried using rotary evaporator (Buchi Corporation, New Castle, DE, United States). The process was repeated three times to maximize extraction. Quantification of alkaloids was performed using a modified method reported by Sharma et al. (2019) with a Waters Xevo TQ-S Micro triple quadrupole mass spectrometer detector connected to Acquity Class I UPLC (Milford, MA, United States). Capillary voltage and source temperature were set to 500 V and 150°C, respectively. A gradient method using a mobile phase consisting of aqueous ammonium acetate buffer (pH 3.5, 10 mM, pump A) and acetonitrile (pump B) was used in combination with Acquity BEH C18 column (1.2 × 100 mm, 1.7 μm) to achieve the chromatographic separation and good peak shape. Flow rate of the mobile phase was 0.35 ml/min and gradient was started with 80% flow through pump A and it was maintained up to 0.5 min. Composition of pump A was decreased linearly to 68% up to 2.2 min and it was further decreased to 62% up to 3.5 min. The composition of pump A was increased to 80% up to 3.6 min and maintained till 4.0 min. Temperature of column oven and autosampler was maintained at 50 and 10°C, respectively.

### Experimental Design and Data Analysis

The experiment was conducted using a complete randomized design with four treatments and 17 replicates. Each plant was regarded as an experimental unit, and within the experimental unit, each leaf sample was a subsample. Bi-weekly alkaloid measurement data for each treatment was pooled together to account for environmental variations over the period of the experiment. Statistical analysis of plant growth data and alkaloid concentrations were conducted using a restricted maximum likelihood mixed model analysis in JMP^®^ Pro 13 (SAS Institute, Inc., Cary, NC, United States) and SAS (SAS Institute, Inc., Cary, NC, United States) with *post hoc* mean separation tests performed using Tukey’s honest significant difference test by fertilization application treatment with treatment combination replicates (*n* = 17) defined as the random error term. Relationships between extracted leaf chlorophyll concentrations and SPAD measurement values were determined using linear regression analysis in JMP^®^ Pro 13. Statistical tests were considered significant if *P* ≤ 0.05.

## Results

### Greenhouse Environmental Conditions

The average daily greenhouse temperature (±SD) was 23.3 ± 5.1°C throughout the experiment. Average relative humidity (±SD) was 78.5 ± 15.7% and daily light integral ranged from 15.2 to 31.4 mol⋅m^–2^⋅d^–1^.

### Alkaloid Content

Leaf alkaloid concentrations as influenced by nutrient application treatment and time following fertilizer application were measured and recorded (Tables 1, 2). 7-Hydroxymitragynine, was below the lower limit of quantification in all samples throughout the study. Mitragynine was detected in 43% of sampled leaves but was below the lower limit of quantification at the beginning of the experiment and was not influenced by the imposed fertilization treatments overall (Tables 1, 2). Similarly, corynoxine was below the lower limit of quantification until late October (Table 1). In general, fertilization did not affect the average paynantheine, speciociliatine, mitraphylline, and corynoxine concentration per leaf dry mass (Table 2). When evaluated by date, paynantheine was below the lower limit of quantification at the beginning of the study but generally above detection limits thereafter (Table 1). Paynantheine concentrations were significant and greatest amongst leaves of trees that received a high rate of fertilizer on the last sampling date, December 10, 2018. Fertilizer application rate influenced speciociliatine concentrations on only one sampling date (November 13, 2018; Table 1) where inverse relationships were observed between speciociliatine and fertilizer rate.

**TABLE 1:** Average alkaloid concentration (±SE) in leaf extract of kratom in response to no (control), low, medium, and high fertilizer rates on individual sampling date.

**Alkaloids**	**Treatment**	**9/14/2018**	**10/1/2018**	**10/16/2018**	**10/29/2018**	**11/13/2018**	**11/26/2018**	**12/10/2018**
Mitragynine	Control	Below LLOQ^*z*^	Below LLOQ	Below LLOQ	0.018 ± 0.005^y^	0.030 ± 0.010	0.016 ± 0.001	0.015 ± 0.000
	Low	Below LLOQ	Below LLOQ	Below LLOQ	0.013 ± 0.002	Below LLOQ	0.017 ± 0.001	0.014 ± 0.001
	Medium	Below LLOQ	Below LLOQ	Below LLOQ	0.015 ± 0.001	0.017 ± 0.003	Below LLOQ	0.019 ± 0.004
	High	Below LLOQ	Below LLOQ	Below LLOQ	0.016 ± 0.003	0.017 ± 0.002	Below LLOQ	0.024 ± 0.002
Speciogynine	Control	0.092 ± 0.020	0.105 ± 0.003 b^x^	0.083 ± 0.005	0.085 ± 0.016	0.060 ± 0.012	0.065 ± 0.006	0.093 ± 0.012
	Low	0.114 ± 0.005	0.188 ± 0.023 a	0.093 ± 0.013	0.093 ± 0.027	0.073 ± 0.010	0.075 ± 0.006	0.130 ± 0.020
	Medium	0.138 ± 0.013	0.168 ± 0.017 ab	0.100 0.026	0.083 ± 0.011	0.140 ± 0.024	0.095 ± 0.009	0.108 ± 0.021
	High	0.132 ± 0.015	0.140 ± 0.007 ab	0.108 ± 0.014	0.065 ± 0.012	0.115 ± 0.021	0.100 ± 0.012	0.095 ± 0.009
Paynantheine	Control	Below LLOQ	0.020 ± 0.000	Below LLOQ	0.023 ± 0.005	0.028 ± 0.005	0.022 ± 0.003	0.024 ± 0.003 b
	Low	Below LLOQ	0.03 ± 0.000	0.020 ± 0.000	0.015 ± 0.003	0.015 ± 0.003	0.023 ± 0.003	0.020 ± 0.002 b
	Medium	Below LLOQ	0.025 ± 0.003	0.020 ± 0.010	0.013 ± 0.003	0.018 ± 0.003	0.020 ± 0.000	0.021 ± 0.002 b
	High	Below LLOQ	0.023 ± 0.002	0.015 ± 0.01	0.020 ± 0.003	0.024 ± 0.004	0.021 ± 0.003	0.034 ± 0.004 a
Speciociliatine	Control	0.028 ± 0.000	0.026 ± 0.005	0.028 ± 0.003	0.025 ± 0.005	0.035 ± 0.004 a	0.023 ± 0.002	0.032 ± 0.006
	Low	0.029 ± 0.004	0.031 ± 0.004	0.025 ± 0.003	0.015 ± 0.003	0.021 ± 0.001 b	0.029 ± 0.003	0.025 ± 0.002
	Medium	0.027 ± 0.000	0.025 ± 0.003	0.027 ± 0.005	0.016 ± 0.002	0.025 ± 0.004 ab	0.026 ± 0.002	0.025 ± 0.006
	High	0.024 ± 0.002	0.023 ± 0.001	0.020 ± 0.001	0.025 ± 0.004	0.023 ± 0.003 ab	0.029 ± 0.004	0.040 ± 0.002
Mitraphylline	Control	0.116 ± 0.020	0.128 ± 0.008	0.113 ± 0.011	0.130 ± 0.019	0.113 ± 0.017	0.120 ± 0.012	0.150 ± 0.014
	Low	0.128 ± 0.005	0.178 ± 0.015	0.118 ± 0.013	0.095 ± 0.022	0.105 ± 0.014	0.105 ± 0.010	0.140 ± 0.015
	Medium	0.136 ± 0.012	0.163 ± 0.013	0.108 ± 0.019	0.080 ± 0.009	0.133 ± 0.021	0.100 ± 0.009	0.113 ± 0.018
	High	0.134 ± 0.004	0.138 ± 0.005	0.105 ± 0.012	0.063 ± 0.013	0.110 ± 0.019	0.108 ± 0.014	0.095 ± 0.006
Corynantheidine	Control	0.070 ± 0.010	0.075 ± 0.01	0.068 ± 0.006	0.070 ± 0.013	0.065 ± 0.013	0.060 ± 0.006	0.083 ± 0.013
	Low	0.092 ± 0.011	0.145 ± 0.029	0.080 ± 0.011	0.050 ± 0.007	0.085 ± 0.010	0.090 ± 0.012	0.128 ± 0.017
	Medium	0.095 ± 0.005	0.108 ± 0.018	0.078 ± 0.015	0.048 ± 0.008	0.073 ± 0.019	0.100 ± 0.010	0.090 ± 0.018
	High	0.092 ± 0.006	0.108 ± 0.019	0.068 ± 0.011	0.030 ± 0.007	0.063 ± 0.013	0.078 ± 0.015	0.075 ± 0.010
Isocorynantheidine	Control	0.096 ± 0.017	0.120 ± 0.015	0.100 ± 0.011	0.090 ± 0.015	0.080 ± 0.016	0.068 ± 0.007	0.098 ± 0.015
	Low	0.140 ± 0.021	0.185 ± 0.025	0.138 ± 0.013	0.068 ± 0.014	0.088 ± 0.014	0.085 ± 0.013	0.125 ± 0.016
	Medium	0.126 ± 0.017	0.168 ± 0.021	0.120 ± 0.023	0.065 ± 0.010	0.108 ± 0.022	0.103 ± 0.014	0.088 ± 0.014
	High	0.120 ± 0.004	0.15 ± 0.015	0.123 ± 0.014	0.050 ± 0.007	0.078 ± 0.015	0.075 ± 0.010	0.070 ± 0.007
Corynoxine	Control	Below LLOQ	Below LLOQ	Below LLOQ	0.019 ± 0.006	0.029 ± 0.003	0.017 ± 0.002	0.024 ± 0.000
	Low	Below LLOQ	Below LLOQ	Below LLOQ	0.010 ± 0.001	0.015 ± 0.002	0.020 ± 0.002	0.012 ± 0.000
	Medium	Below LLOQ	Below LLOQ	Below LLOQ	0.011 ± 0.003	0.020 ± 0.004	0.012 ± 0.000	0.021 ± 0.003
	High	Below LLOQ	Below LLOQ	Below LLOQ	0.014 ± 0.004	0.022 ± 0.002	0.018 ± 0.002	0.025 ± 0.001

*Data were pooled from four replicates and means with the same letter are not statistically different by Tukey’s honest significant difference test at *P* ≤ 0.05.*

*^*z*^LLOQ = Lower limit of quantitation.*

*^*y*^Non-significant by Tukey’s HSD test at *P* ≤ 0.05.*

*^*x*^Means sharing the same letter are not statistically different according to Tukey’s honestly significant difference (HSD) test at *P* ≤ 0.05.*

**TABLE 2:** Average alkaloid concentration (±SE) in leaf extract of kratom in response to no (control), low, medium, and high fertilizer rates.

**Treatment**	**Alkaloid concentration per leaf dry mass (%w/w)**								
	**Mitragynine**	**Speciogynine**	**Paynantheine**	**Speciociliatine**	**Mitraphylline**	**Corynantheidine**	**Isocorynantheidine**	**Corynoxine**
Control	0.018 ± 0.003	0.083 ± 0.005 b	0.024 ± 0.002	0.028 ± 0.002	0.124 ± 0.006	0.07 ± 0.004 b	0.093 ± 0.006 b	0.022 ± 0.002
Low	0.016 ± 0.002	0.109 ± 0.009 ab	0.020 ± 0.001	0.025 ± 0.001	0.124 ± 0.007	0.1 ± 0.007 a	0.119 ± 0.009 a	0.015 ± 0.002
Medium	0.018 ± 0.001	0.119 ± 0.008 a	0.020 ± 0.001	0.024 ± 0.002	0.119 ± 0.007	0.084 ± 0.006 ab	0.111 ± 0.008 ab	0.017 ± 0.002
High	0.018 ± 0.002	0.109 ± 0.006 ab	0.023 ± 0.001	0.027 ± 0.002	0.108 ± 0.006	0.074 ± 0.006 b	0.096 ± 0.007 b	0.020 ± 0.001

*Data were pooled from four replicates bi-weekly for 4 months and means with the same letter are not statistically different by Tukey’s honest significant difference test at *P* ≤ 0.05.*

**FIGURE 2:**
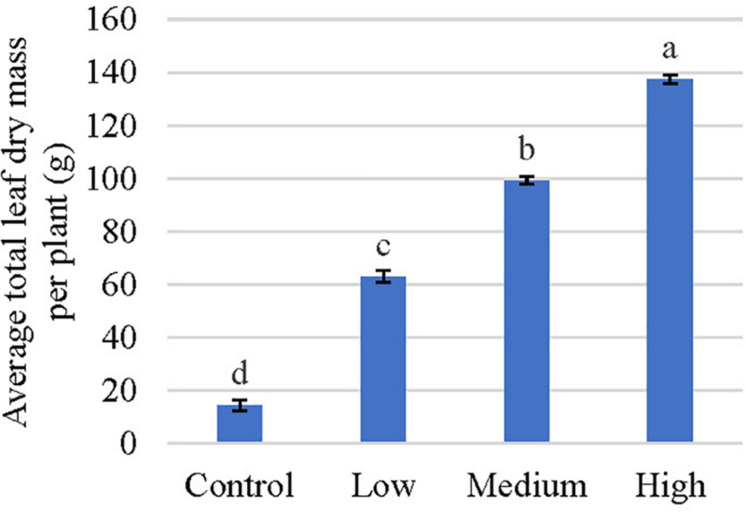
Average total leaf dry mass per plant of kratom in response to no (control), low, medium, and high fertilizer rates. Data were pooled from four replicates and means with the same letter are not statistically different by Tukey’s honest significant difference test at *P* ≤ 0.05. Error bars indicate the standard error.

Given no consistent temporal trends were observed for alkaloids quantified in this study, data was pooled together to increase statistical power for analysis of overall alkaloidal trends (Table 2). Average concentrations of speciogynine, corynantheidine, and isocorynantheidine were influenced by fertilizer application rate (Table 2). Although fertilization did not affect corynantheidine and isocorynantheidine concentration on individual sampling dates (Table 1), their levels were greatest in leaves of trees that received low rates of fertilizer, with concentrations approximately 43 and 28% greater than the control, respectively (Tables 1, 2). Speciogynine concentrations for each treatment were similar on each sampling date except on October 1, 2018 where plants that received a low fertilization rate had a greater concentration compared to plants that received no fertilizer (Table 1). However, when data was pooled, speciogynine was greatest in leaves of trees that received medium rates of fertilizer at 43% greater than the control. Using mean alkaloid concentration data (Table 2) along with total leaf biomass (Figure 2), total alkaloid content within trees was estimated and observed to vary significantly among fertilizer treatments given large differences in total leaf dry mass (Table 3). Greatest alkaloidal mass was estimated to occur in trees that received high fertilizer rates.

**TABLE 3:** Total estimated alkaloid concentration (±SE) in kratom trees in response to no (control), low, medium, and high fertilizer rates.

**Treatment**	**Total alkaloid content per plant (%w/w)**								
	**Mitragynine**	**Speciogynine**	**Paynantheine**	**Speciociliatine**	**Mitraphylline**	**Corynantheidine**	**Isocorynantheidine**	**Corynoxine**
Control	0.27 ± 0.03 d	1.20 ± 0.07 d	0.35 ± 0.03 d	0.41 ± 0.03 d	1.78 ± 0.08 d	1.01 ± 0.06 c	1.35 ± 0.08 d	0.32 ± 0.03 c
Low	0.94 ± 0.06 c	6.96 ± 0.56 c	1.26 ± 0.09 c	1.57 ± 0.08 c	7.82 ± 0.43 c	5.99 ± 0.46 b	7.49 ± 0.58 c	0.94 ± 0.11 bc
Medium	1.62 ± 0.13 b	11.77 ± 0.82 b	2.01 ± 0.11 b	2.37 ± 0.15 b	11.80 ± 0.67 b	8.32 ± 0.59 a	10.99 ± 0.82 b	1.71 ± 0.21 b
High	2.58 ± 0.23 a	14.92 ± 0.89 a	3.2 ± 0.20 a	3.64 ± 0.22 a	14.87 ± 0.79 a	10.23 ± 0.80 a	13.18 ± 0.96 a	2.76 ± 0.20 a

*Data were pooled from four replicates bi-weekly for 4 months and means with the same letter are not statistically different by Tukey’s honest significant difference test at *P* ≤ 0.05.*

### Plant Growth Indicators

**FIGURE 3:**
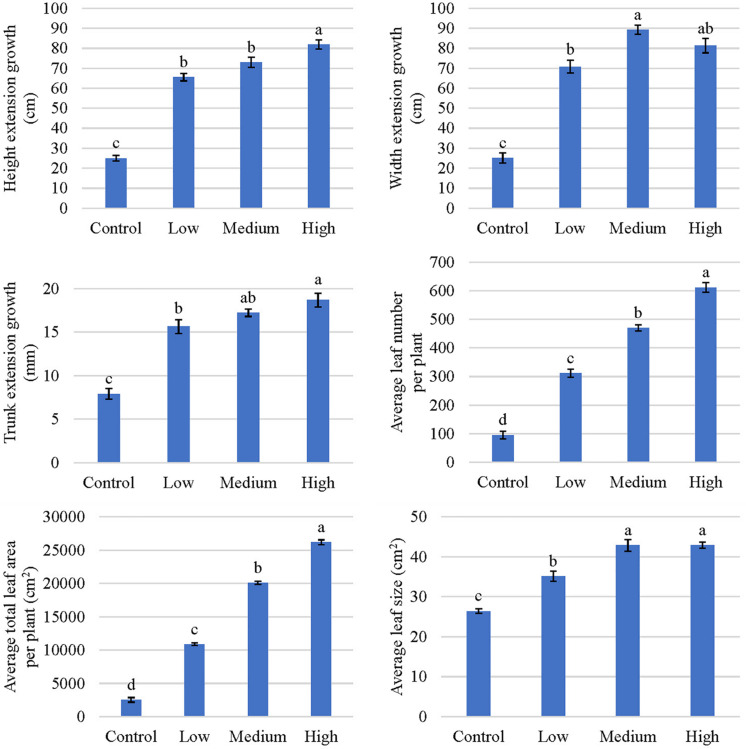
Plant growth indicators of kratom in response to no (control), low, medium, and high fertilizer rates. Leaf number included leaves ≥2 cm. Data were pooled from 17 replicates for height, width, and trunk extension growth and four replicates for average leaf number per plant, average total leaf area per plant, and average leaf size. Means with the same letter are not statistically different by Tukey’s honest significant difference test at *P* ≤ 0.05. Error bars indicate the standard error.

Tree height, width, and trunk growth increased as the fertilizer application rate increased, with the tallest and widest plants occurring in response to the high fertilization treatment (Figure 3). Extension of plant height increased by 162, 192, and 228%, in response to low, medium, and high fertilization, respectively, when compared to trees in the control (non-fertilized) group. Similarly, the average extension of plant width increased 46, 64, and 56 cm, respectively, in response to low, medium, and high fertilization treatments. Trunk diameter increased 2-, 2.2-, and 2.4-fold in response to low, medium, and high fertilizer application rates, respectively (Figure 3).

Trees that received fertilizer had an average increase in leaf number of between 217 and 517 (Figure 3). Total leaf area increased by approximately 4-, 8-, and 10- fold, respectively, in response to low, medium, and high fertilizer applications. Similarly, average leaf size increased 9 to 16 cm^2^ in response to fertilizer applications; however, no differences were observed in average leaf size between the medium and high fertilizer treatments. Total leaf dry mass increased as the fertilizer rate increased, with the greatest leaf mass recorded in response to the high fertilizer treatment (Figure 2). An average of 123 g (10-fold) difference in leaf dry mass per plant was observed between trees that received high fertilizer application rates and the control.

### SPAD and Chlorophyll Concentration

**FIGURE 4:**
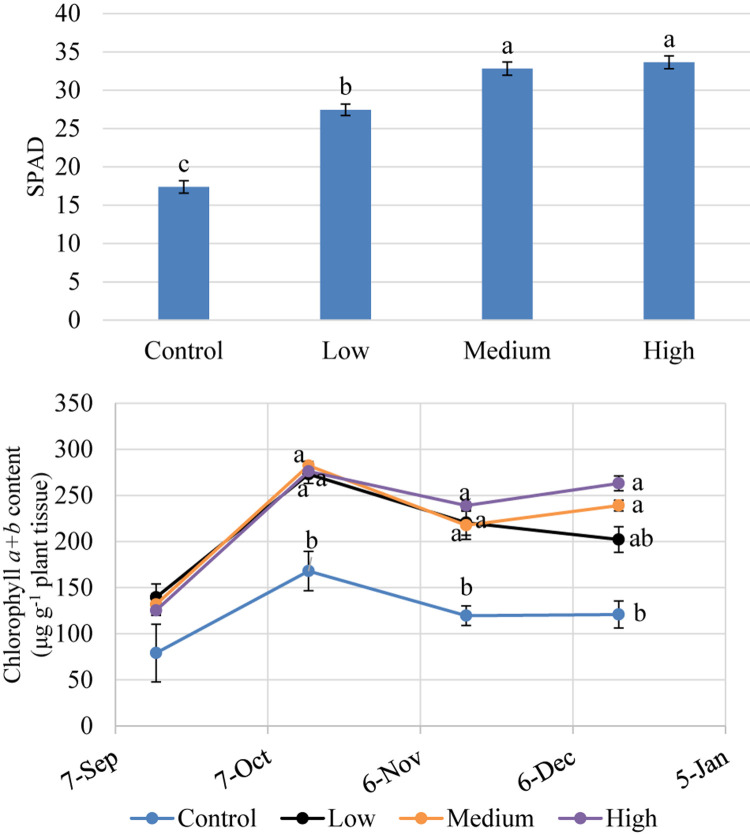
SPAD index values and extracted leaf chlorophyll concentration of kratom in response to no (control), low, medium, and high fertilizer rates. SPAD data were pooled from 12 replicates and chlorophyll *a* + *b* content data were pooled from three replicates each month. Means with the same letter are not statistically different by Tukey’s honest significant difference test at *P* ≤ 0.05. Error bars indicate the standard error.

**FIGURE 5:**
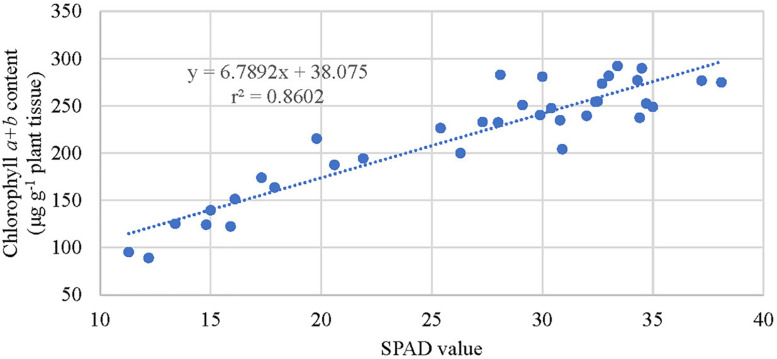
Relationship between extracted leaf chlorophyll concentration of kratom and SPAD index values cultivated between October and December 2018 in response to no (control), low, medium, and high fertilizer rates. SPAD values and chlorophyll content data were pooled from 12 replicates.

Extracted chlorophyll concentrations were similar among trees (Figure 4) at the initiation of the experiment (September 2018). Between October and December 2018, trees that received fertilizer had significantly greater chlorophyll concentrations than the control trees but were not significantly different among fertilizer treatment rates. Fertilizer increased SPAD values by 57 to 93% over the control. SPAD values were highest in trees that received medium and high fertilizer application rates (Figure 4). Overall, chlorophyll concentrations for trees receiving fertilizer increased by 67 to 118% over the control. SPAD values correlated well (*r*^2^ = 0.86) with extracted chlorophyll concentrations among trees (Figure 5) between October and December 2018.

## Discussion

Regardless of fertilizer application rate imposed in our study, mitragynine concentrations were not significantly influenced by treatment (Tables 1, 2). Overall, mitragynine concentrations were an order of magnitude lower than those reported in commercial kratom products (Sharma et al., 2019) and approximately 4.5-fold below the lowest reported concentrations observed in collected commercial bulk leaf samples (Kikura-Hanajiri et al., 2009). Although mitragynine has long been considered the dominant alkaloid by alkaloidal fraction, concentrations can vary greatly by source. Takayama et al. (1998) observed mitragynine to vary between 12 and 66% (%w/w) in leaves collected from Malaysia and Thailand, respectively. A related study examining mitragynine presence in leaves collected from a diverse set of environments in the Philippines only detected mitragynine in 38% of collected samples (Uy et al., 2020). In addition to spatial variation, temporal variation in mitragynine concentrations have been reported to vary by approximately 20% (Shellard et al., 1978a). High geographical variance and even chemical races among species were likely the sources of both seasonal and batch-to-batch variability. Plant maturity is thought to be an influential factor of mitragynine content (Shellard et al., 1978b). However, Shellard’s study on plant maturity-mitragynine concentration relationships included only three kratom trees germinated from seed mistakenly fertilized by a different Mitragyna species; thus, a lack of experimental robustness confounds reliable interpretation and application of reported results.

Some effects of kratom consumption, as reported by users, may be mistakenly attributed to the presence or absence of mitragynine. A cross-section survey of Malaysian kratom users indicated that medical efficacy of kratom tea consumption was maximized when consumed within 24 h of brewing (Singh et al., 2019). Effectiveness of kratom tea was reported to decrease when consumed 48 and 72 h after brewing. However, laboratory analysis of the kratom tea found no differences in mitragynine concentrations throughout the 72-h period (Singh et al., 2019). Thus, results suggest changes in compounds other than mitragynine may play a much more significant role in reported medical effectiveness of kratom than what is currently understood. Coupled with a new understanding of the functioning of kratom’s minor alkaloids (Obeng et al., 2020), mitragynine may not be as important as once thought for specific medical applications.

7-Hydroxymitragynine was not detected in any of our samples. Low levels of mitragynine present in the sampled leaves were therefore not in response to the hydroxylation of mitragynine to 7-hydroxymitragyine. Low levels of mitragynine and no detection of 7-hydroxymitragyine among leaves in our study suggests low substance abuse liability as these two compounds are believed to be responsible for the potent μ-opioid receptor agonist activity associated with consumption of kratom.

Despite a long history of cultivation in southeast Asia, kratom production practices are largely undocumented and thus confound application of established management practices that aim to maximize plant growth and alkaloid synthesis. As a modern specialty crop in North America, information on relationships between horticultural practices and plant performance is limited but critical to support development of a newly emerging kratom production industry. Moreover, knowledge of relationships between common management practices and plant response is foundational to future scientific investigations on this unique specialty crop. Additional research examining relationships between production environment, plant growth, and alkaloid synthesis is necessary to further understand kratom and to develop reliable, efficient management practices.

Overall, limited temporal relationships were observed for leaf alkaloid concentrations with the progression of time (Table 1). Given a study duration of 4 months using a fertilizer with a linear release profile, future research examining synthesis of leaf alkaloids over a longer duration of time, such as an entire season, or with fertilizers possessing distinctly different nutrient release and availability characteristics may elucidate relationships not observed in this study. Although concentration slightly varied on each sampling date, fertilization rate overall did not influence the minor alkaloids paynantheine, mitraphylline, speciociliatine, and corynoxine (Tables 1, 2). Speciociliatine and paynantheine content in our samples were lower than values reported in powdered commercial kratom samples (Sharma et al., 2019). The range of corynantheidine concentrations in our samples, however, were similar to those reported in commercial kratom products. Isocorynantheidine content, regardless of fertilizer treatment, was greater than those reported in sampled commercial kratom products with the exception of “Supernatural sun red label” (Sharma et al., 2019). Corynantheidine and isocorynantheidine function as opioid antagonists and a muscle relaxant. Coupled with low levels of mitragynine and the absence of 7-hydroxymitragynine, tea made from our sampled leaves would likely present different activity and therapeutic effects than what would be expected from commercial kratom products analyzed by Sharma et al. (2019).

Interestingly, speciogynine, corynantheidine, and isocorynantheidine concentrations were highest in plants that received low or medium fertilizer applications (Table 2). Although the effect of treatment was not significant on each sampling date (Table 1), the overall influence was significant when data was pooled together due to the increase of data sample size (Table 2). Results suggested low nutrient supply conditions favor allocation of nitrogen for synthesis of these select metabolites as long as they are not below critically low levels (control treatment) that inhibit both primary and secondary metabolism. Similar trends in primary and secondary metabolism of indole alkaloids in response to nutrient availability were observed for the tropical ethnobotanical tree *Tabernaemontana pachysiphon* (Höft et al., 1996).

Nitrogen, potassium, and phosphorus may separately affect indole alkaloid synthesis, although information is limited. Intracellular concentration of ajmalicine and serpentine decreased with increased concentration of phosphate in cell suspension cultures of periwinkle (Knobloch and Berlin, 1983). Conversely, concentrations of vinblastine in leaves and catharanthine within leaves and roots of periwinkle significantly increased when treated with 80 and 100 mM potassium soluble fertilizer (KNO_3_) compared to non-treated plants (Chang et al., 2014). Chemical forms of applied nutrients may also influence synthesis of alkaloids. For example, Misra and Gupta (2006) indicated that alkaloid content in both leaf pairs and roots of periwinkle was maximal in response to application of NO_3_^–^; however, alkaloid content was not influenced by application of NH_4_^+^. In our study, we applied mostly equivalent amounts of ammoniacal and nitrate nitrogen and did not observe a significant increase in the concentration of most alkaloids tested despite varying fertilization rates. Individual macro- and micro-nutrients may influence the synthesis of alkaloids within leaves of kratom and thus further research is warranted.

Plant growth parameters (height, width, trunk, leaf number and area, and total dry mass) increased significantly in response to increased fertilizer application rates. Even at the highest fertilizer application rate, nitrogen toxicity was not observed in our study. Fertilizer did not increase mitragynine concentrations per leaf dry mass. However, it did influence the total harvestable biomass. Thus, total harvestable mitragynine per plant increased by 2-, 5-, and 7-fold in response to low, medium, and high fertilization rates, respectively, compared to those given no fertilizer (Table 3). Similarly, high fertilizer application rates resulted in a 9- to 12-fold increase in total harvestable mass of speciogynine, paynantheine, speciociliatine, mitraphylline, corynantheidine, isocorynantheidine, and corynoxine compared to trees that received no fertilizer. Although greater amounts of plant nitrogen were likely allocated to secondary metabolism of select minor alkaloids in trees that received low and medium rates of fertilizer, total alkaloidal content per plant was greatest in trees that received high fertilizer application rates due to significantly greater plant biomass in our study. These trends suggested that high application rate of fertilization would be beneficial when extracting and concentrating alkaloids from total harvested biomass is the production goal for concentrated commercial products. Alternatively, low to medium fertilization application rates would be preferential for brewing tea with direct steeping of leaves to achieve a higher water-soluble alkaloid concentrations per leaf dry mass.

Leaf chlorophyll content was reliably estimated using SPAD meter values and the linear model developed in our study (Figure 5). However, chlorophyll and SPAD values were not significantly different among fertility treatments. Thus, relying upon SPAD or chlorophyll values to differentiate between trees that received low, medium, and high fertilizer applications, as defined in this study, would not be possible. Additional research examining a wider range of fertility applications may allow for a differentiation in SPAD response among fertilizer application rates. Regardless, strong correlations between SPAD and leaf chlorophyll content allow for timely, simple, non-destructive estimation of kratom nutrient status. This rapid test can help in the diagnosis of nutrient deficiencies and allow a timely adjustment of fertilization programs aimed to maximize the production of desired alkaloids in kratom trees.

## Conclusion

As a new emerging crop, relevant information on kratom cultivation is limited despite its long cultivation history in southeast Asia. Documented relationships between kratom growth and alkaloid synthesis have until now been unavailable. For commercial cultivation of kratom to be successful, a greater understanding of relationships between kratom management practices and plant growth and performance are needed. In this study, higher fertility rates greatly promoted plant growth by increasing plant height and width, trunk diameter, leaf number, leaf area, and leaf dry mass. Mitragynine and 7-hydroxymitragyine were detected at low levels and below the lower limit of quantification, respectively. Low to medium rates of fertilizer maximized the concentration of speciogynine, corynantheidine, and isocorynantheidine per leaf dry mass, suggesting a promotion of nitrogen allocation for secondary metabolism and thus their synthesis under such condition, but fertilization rate had little influence on other alkaloids. Higher fertility rate resulted in a higher SPAD values and chlorophyll concentration in fresh leaves. A linear model between rapid, non-destructive chlorophyll evaluation index (SPAD) and destructively extracted chlorophyll concentrations was developed to help diagnose nutrient deficiencies and allow for timely and reliable method to adjust fertility programs to aid in enhanced management of kratom cultivation. Findings suggest either kratom trees utilized in this study were likely of a different genotype than those used as a source for commercial kratom products or another key environmental factor responsible for mitragynine and/or other alkaloids synthesis was not present in our study. Additional research identifying other key factors responsible for alkaloid synthesis and kratom growth are warranted.

## Data Availability Statement

The raw data supporting the conclusions of this article will be made available by the authors, without undue reservation.

## Author Contributions

MZ: conceptualization, methodology, validation, formal analysis, investigation, data curation, writing–original draft, writing–review and editing, visualization. AS: methodology, formal analysis, investigation, writing–review and editing. FL: methodology, investigation, writing–review and editing. BA: methodology, supervision. RK: writing–review and editing, supervision, project administration. CM: resources, writing–review and editing, supervision, project administration. BP: conceptualization, methodology, validation, formal analysis, resources, writing–review and editing, visualization, supervision, project administration, funding acquisition. All authors contributed to the article and approved the submitted version.

## Conflict of Interest

The authors declare that the research was conducted in the absence of any commercial or financial relationships that could be construed as a potential conflict of interest.
